# Evaluation of a novel reprocessed bCPAP system on sepsis rates among preterm neonates with respiratory distress: a randomized controlled trial

**DOI:** 10.3389/fped.2025.1569437

**Published:** 2025-05-19

**Authors:** Sarah Badin, Navid Roodaki, Daisy Evangeline C. Garcia, Rochelle Abila-Cariaga, Thomas F. Burke

**Affiliations:** ^1^ Department of Implementation, Vayu Global Health Foundation, Medford, MA, United States; ^2^ Department of Emergency Medicine, Massachusetts General Hospital, Boston, MA, United States; ^3^ Department of Pediatrics, Ilocos Training and Regional Medical Center, San Fernando, Philippines; ^4^ Department of Pediatrics, Mariano Marcos State University, College of Medicine Ilocos Norte, Batac, Philippines; ^5^ Department of Emergency Medicine, Harvard Medical School, Boston, MA, United States; ^6^ Harvard T.H. Chan School of Public Health, Boston, MA, United States

**Keywords:** neonate, bCPAP, reuse, neonatal sepsis, respiratory distress

## Abstract

**Introduction:**

Bubble CPAP (bCPAP) is highly effective in the treatment of respiratory distress syndrome of prematurity and other causes of newborn respiratory insufficiency. To overcome barriers to bCPAP access a novel system was developed that is designed to be cleaned, disinfected, and reused. This study evaluated whether use of reprocessed bCPAP systems increases the rate of sepsis in neonates.

**Methods:**

A *post hoc* analysis of a single-center randomized controlled trial (registration no. NCT06082674) was conducted that compared mechanical ventilator driven CPAP devices (MV-CPAP) with single-use circuits to reusable bCPAP systems that were cleaned and disinfected after each use. The primary outcome was a composite of treatment escalation or death.

**Results:**

Seventy-five neonates were randomized to the two CPAP treatment arms. No significant differences in death (5 vs. 4), escalation of care (10 vs. 9), and the composite outcome (OR = 0.84; 95% CI: 0.30–2.35, *p* = 0.743) were detected in the MV-CPAP and bCPAP groups respectively. There were no clinically significant differences in any of the secondary outcomes.

**Discussion:**

Use of a reprocessed bCPAP system designed to increase global access to CPAP did not increase rates of neonatal sepsis.

**Clinical Trial Registration:**

ClinicalTrials.gov, identifier, (NCT06082674).

## 1 Introduction

Approximately five million children under age five died in 2022 worldwide, two million of whom died in the first 28 days of life (1, 2). Over 95% of these deaths occurred in low-and-middle income countries (LMICs) (3, 4). Preterm birth complications, such as Respiratory Distress Syndrome (RDS), pneumonia, neonatal sepsis, and birth asphyxia are among the leading causes of morbidity and mortality during the neonatal period (1, 2).

Continuous Positive Airway Pressure (CPAP) is a proven effective treatment for respiratory distress in neonates (5, 6) and was formally recommended by WHO in November of 2022 (7). CPAP has been shown to improve survival and decrease the need for surfactant and mechanical ventilation (6, 8). However, access to quality CPAP devices is limited in LMICs (4) due to cost and the lack of bioengineering, compressed air, functional supply chain, and reliable electricity (4, 9, 10).

The Vayu bubble CPAP (bCPAP) system (Vayu Global Health Innovations, Public Benefit Corporation, Boston, MA, USA) was designed to overcome barriers to global CPAP access. The system is low-cost and has been shown to significantly improve the work of breathing, oxygenation, and survival of neonates in respiratory distress (11–15). It provides blended oxygen and precise mixed gas flow rates, pressures, and humidification without the need for electricity, compressed air, or advanced bioengineering support. Additionally, this novel system was designed with components that can be cleaned and disinfected for long-term repeated use (13, 15).

In neonatal intensive care units (NICUs), where patients are particularly vulnerable, sepsis is a significant contributor to mortality (1, 2, 16). Sepsis impacts approximately 1.3 million neonates annually and is the third leading cause of death in that age group (1, 2, 17). Late-onset sepsis, which occurs at least 3 days after birth is primarily due to nosocomial infections and is more prevalent in preterm and low-birth weight neonates (18). Nosocomial infections are more common when treatment modalities include catheters placed in veins, arteries, and the bladder as well as with use of invasive ventilation (18, 19). Although reusable medical equipment may improve access to life-saving devices, they also may increase the risk of infections (20, 21). Strict adherence to infection control practices and disinfection protocols are essential to preventing sepsis in NICUs (18, 19).

Independent evaluation of the cleaning and disinfection instructions for the Vayu bCPAP system was performed by Eurofins Lancaster Laboratories (Lancaster, PA, USA). While tests showed that the protocol meets stringent infection control safety standards, they did not take into account nor evaluate the human factors of real-world use. In this study we compared neonates randomized to mechanical ventilator driven CPAP (MV-CPAP) devices with single use consumables to those randomized to reprocessed Vayu bCPAP systems and consumables to evaluate for the risk of sepsis.

## 2 Materials and methods

### 2.1 Study design

We conducted a *post hoc* analysis of data from a randomized controlled trial performed in the NICU of the Ilocos Training and Regional Medical Center (ITRMC) in the Philippines, from May until October 2022. The research protocol was approved by the Ilocos Technical Review Board and Research Ethics Committee (ITRMC-REC-2022-11) and enrolled in clinicaltrial.gov (PRS number NCT06082674). Mothers for whom preterm deliveries were anticipated were approached for consent prior to delivery. Written informed consent was obtained from the parents of all enrolled neonates. Parents and guardians were informed about their right to withdraw from the study at any given time.

### 2.2 Study setting

ITRMC is a referral facility located in San Fernando, La Union in the northern region of the Philippines. It houses a 25-bed NICU that can accommodate up to 50 neonates. During the day, the NICU is staffed with four registered nurses, one nursing attendant, one consultant neonatologist, and three postgraduate trainees. In the evening and overnight, four registered nurses, one nursing attendant, and one postgraduate trainee are in the NICU with a consultant level neonatologist available by telephone. The NICU offers advanced care to neonates including mechanical ventilation, nasal intermittent positive pressure ventilation (NIPPV), CPAP, surfactant administration, umbilical catheters, antibiotic treatment, and point of care ultrasound (POCUS). The unit includes incubators, radiant warmers, pulse oximeters, monitors, and a designated portable x-ray. There is a dedicated utility room for cleaning and disinfecting reusable bCPAP systems and other equipment.

### 2.3 Study population

Preterm neonates (< 37 weeks) for whom written consent was obtained prior to delivery and who were treated with CPAP within 6 hours of delivery were screened for study eligibility and randomization. Neonates who (1) underwent resuscitation (bag mask ventilation, chest compression, or intubation at birth), (2) had APGAR scores at 1 and 5 minutes <7, or (3) had congenital anomalies that might interfere with CPAP therapy were excluded from the study. All patients enrolled in the original trial were included in the analysis.

### 2.4 Study procedure and intervention

Eligible neonates were randomly assigned to CPAP treatment with a mechanical ventilator (MV-CPAP; Puritan Bennett 840, Medtronic, Minneapolis, MN, USA) device with single use components or to a cleaned and disinfected Vayu bCPAP system, here-on referred to as a bCPAP system. Neonates randomized to a MV-CPAP device were considered to be the control group and those randomized to a bCPAP system were considered to be the intervention group. Randomization was done by opening sequentially numbered opaque envelopes that randomly assigned the form of CPAP treatment.

CPAP therapy was not available in labor and delivery (L&D) and was always initiated in the NICU. All neonates were placed on continuous cutaneous temperature monitors (Mindray or Draeger), and temperatures were recorded hourly. If available, neonates treated with CPAP were placed in an incubator if their birth weight was less than 1,200 g. Those with a birth weight of 1,200 g or above were placed in a radiant warmer while on CPAP treatment. If the neonate was stable, Kangaroo Mother Care was encouraged although it was not a common practice.

Neonates were weaned off CPAP when respiratory distress improved, pulse oximetry saturations were greater than 90% on an FiO_2_ less than or equal to 30%, and CPAP pressures were less than or equal to 5 cm of water. Neonates were escalated from CPAP to NIPPV or invasive ventilation when while being treated with CPAP, one or more of the following occurred: (1) failure to meet a target saturation of 90%–95% despite an FiO_2_ of 60% (2) two or more apneic episodes that required stimulation and bag-mask ventilation or (3) a Silverman Andersen Respiratory Severity Score (RSS) of >6 despite an FiO_2_ ≥ 60% and a pressure ≥ 6 cm of water.

**Figure 1:**
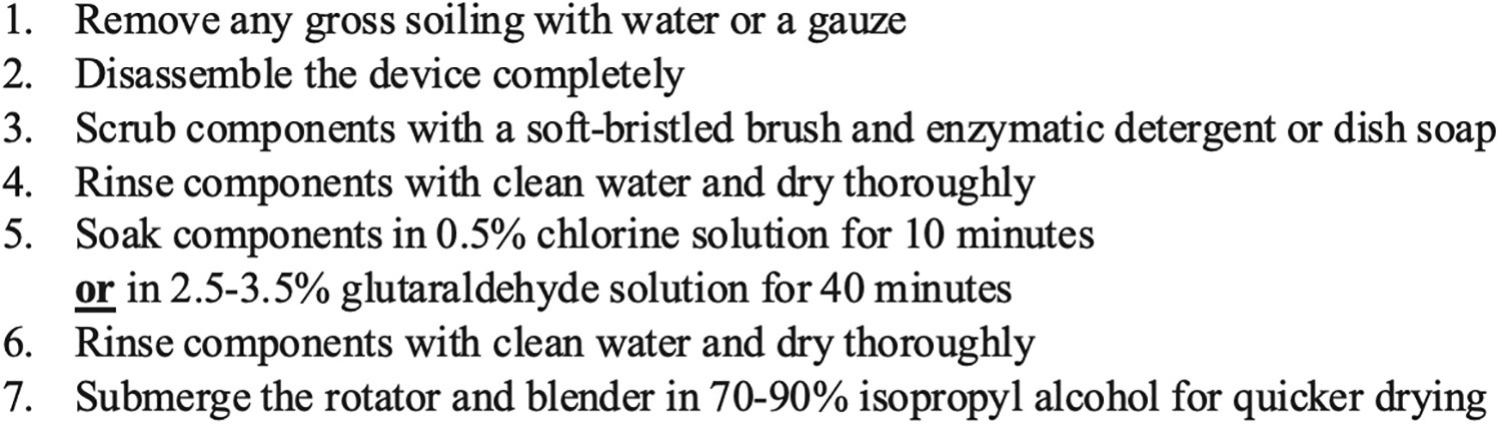
Vayu bCPAP system Instructions for Use - cleaning and disinfection protocol.

**Figure 2:**
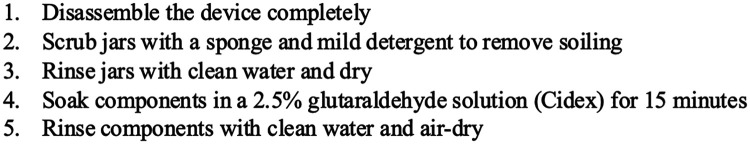
ITRMC modified cleaning and disinfection procedure.

Single use components of the MV-CPAP device included the breathing tubes, nasal cannula, and filters. Components of the bCPAP system that were cleaned and disinfected included the humidifier and pressure generator jars, pressure generator wand, jar lids, warmer bracket, blender clip, blender, filter housing, pressure generator connector, oxygen source adapter, oxygen tube, humidifier tube, breathing tubes, nasal prongs, and safety pins. The validated cleaning and disinfection protocol included in the bCPAP system Instructions for Use is outlined in Figure 1. ITRMC independently modified the cleaning and disinfection protocol as shown in Figure 2. The entire bCPAP system was reprocessed using the ITRMC protocol except for the mustaches, soft loop fasteners, filter disks, and rubber bands which were discarded. The nursing staff were responsible for reprocessing the components.

### 2.5 Data collection and analysis

Gestational age, birth weight, sex, APGAR scores at one and five minutes, and length of time of ruptured membranes were recorded. The RSS and pulse oximetry saturations were captured immediately prior to application of CPAP.

The day of CPAP initiation was considered day 0. Immediately after CPAP was initiated a blood culture was drawn from the umbilical vein and antibiotics (ampicillin and gentamycin) were administered. The FiO_2_ and pressure settings on initiation of CPAP and the number of surfactant doses given were recorded.

A complete blood count (CBC), procalcitonin level, and C-reactive protein (CRP) level were obtained 24 hours after birth (day 1). Additional CBCs were obtained on days 3 and 5 of CPAP treatment. All blood draws (blood cultures, CBCs, procalcitonin, and CRP levels) were obtained through the umbilical vein. The number of days on CPAP, need for treatment escalation, clinical deterioration, and survival to discharge were recorded.

The primary outcome was a composite of death prior to discharge and need for treatment escalation. Secondary outcomes included the following indicators associated with sepsis: clinical deterioration, platelet and white blood cell (WBC) counts on days 3 and 5, temperature instability (defined as <36.5°C or >37.5°C), and the number of days on CPAP treatment.

We assumed that the risk of introducing a nosocomial infection from the device in the control group was 0% and that any increased adverse outcomes potentially associated with sepsis or infection in the intervention group were due to the use of reusable components.

Baseline characteristics were compared between the two study groups using Chi-square for categorical indicators and Student's *t*-test for continuous variables. Binary outcomes in the two groups were assessed with Firth's logistic regression. Continuous outcomes were assessed by linear regression. Analyses were conducted using R version 4.2.1.

## 3 Results

**Figure 3:**
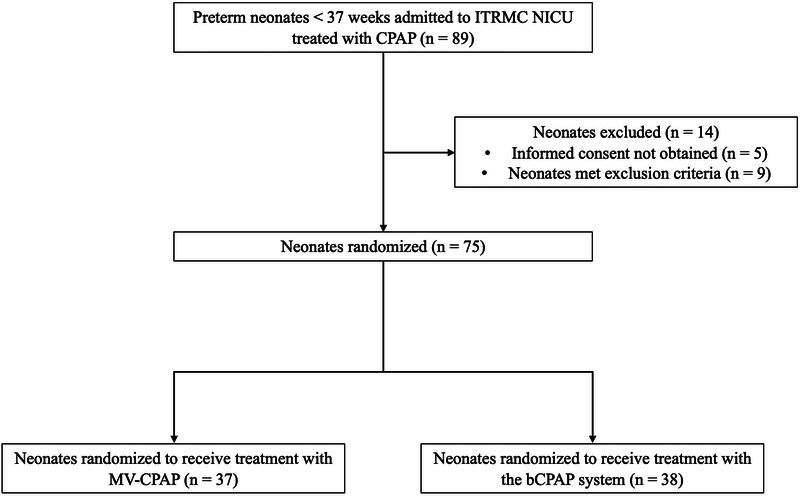
Flowchart of patients recruited in the trial.

A total of 89 preterm neonates were treated with CPAP at ITRMC during the study period, of whom 75 met inclusion criteria and were randomized to receive treatment with either an MV-CPAP device (*n* = 37, 49.3%) or a cleaned and disinfected bCPAP system (*n* = 38, 50.7%). All randomized neonates were included in the final analysis (shown in Figure 3). The median gestational age and weight of enrolled neonates were 32 weeks (IQR: 31–34 weeks) and 1,200 g (1,025–1,400 g), respectively. APGAR scores across the two groups were similar. There were no significant differences in the baseline and clinical characteristics of the two study groups (shown in Table 1).

**Table 1:** Baseline demographic and clinical characteristic.

Characteristic	Overall	MV-CPAP	bCPAP
Sample size, *n* (%)	75 (100.0)	37 (49.3)	38 (50.7)
Sex, *n* (%)			
Female	38 (50.7)	21 (56.8)	17 (44.7)
Male	37 (49.3)	16 (43.2)	21 (55.3)
Gestational age, mean (SD), weeks	32.3 (2.1)	32.4 (1.7)	32.1 (2.4)
Weight, mean (SD), grams	1,266.9 (326.7)	1,311.6 (375.1)	1,223.4 (269.5)
Prolonged rupture of membranes, mean (SD), h	10.3 (6.3)	9.8 (5.7)	10.9 (7.4)
RSS score prior to CPAP initiation, mean (SD)	6.5 (0.5)	6.4 (0.5)	6.6 (0.5)
SpO2 prior to CPAP initiation, mean (SD)	86.6 (4.1)	87.2 (3.6)	86.0 (4.5)
Number of surfactant doses given, mean (SD)	0.7 (0.5)	0.7 (0.5)	0.7 (0.5)
Initial blood culture, *n* (%)			
*No growth*	65 (86.7)	32 (86.5)	33 (86.8)
*Coag. Negative Staph.*	3 (4.0)	1 (2.7)	2 (5.3)
*E. Coli*	1 (1.3)	0 (0.0)	1 (2.6)
*Group B Strep.*	5 (6.7)	3 (8.1)	2 (5.3)
*Staph. Aureus*	1 (1.3)	1 (2.7)	0 (0.0)
Day 1 labs, mean (SD)			
Procalcitonin, mg/ml	7.5 (4.2)	7.2 (4.4)	7.7 (4.2)
CRP, mg/dl	9.9 (5.9)	9.9 (5.7)	9.8 (6.1)
Platelets, × 10^9^/L	242.0 (33.9)	237.6 (37.6)	246.4 (29.8)
WBC count, × 10^9^/L	17.5 (4.5)	17.2 (4.0)	17.8 (5.0)

Out of the 37 neonates in the MV-CPAP treatment group, 7 (19.0%) were escalated to NIPPV and 3 (8.0%) were escalated to invasive ventilation. In the bCPAP treatment group, 6 out of 38 (15.8%) were escalated to NIPPV and 3 (7.9%) were escalated to invasive ventilation. Five out of 37 neonates (13.5%) in the control group and 4 out of 38 (10.5%) neonates in the intervention group died. The composite outcome of death and treatment escalation was similar in both groups (OR = 0.84; 95% CI: 0.30–2.35, *p* = 0.743). Six neonates in each group clinically deteriorated (OR = 0.97; 95% CI: 0.29–3.26, *p* = 0.959). The mean difference in the time spent on CPAP treatment between the intervention and control groups was −0.65 days (95% CI: −1.43–0.13, *p* = 0.100). A summary is shown in Table 2.

**Table 2:** Treatment outcomes.

Outcome	MV-CPAP (*n* = 37)	bCPAP (*n* = 38)	Unadjusted odds ratio (95% CI)
Composite outcome (death or treatment escalation), *n* (%)	10 (27.0)	9 (23.7)	0.84 (0.30, 2.35)
Clinical deterioration, *n* (%)	6 (16.2)	6 (15.8)	0.97 (0.29, 3.26)
Outcome	MV-CPAP (*n* = 37)	bCPAP (*n* = 38)	Unadjusted Linear difference (95% CI)
Duration of CPAP treatment, mean (SD), days	3.8 (2.0)	3.2 (1.4)	−0.65 (−1.43, 0.13)
WBC count day 3, mean (SD), ×10^9^/L	15.4 (5.0)	17.7 (5.1)	2.32 (−0.18, 4.82)
WBC count day 5, mean (SD), ×10^9^/L	15.3 (4.5)	15.6 (4.6)	0.24 (−2.39, 2.87)
Platelet count day 3, mean (SD), ×10^9^/L	224.4 (49.4)	244.9 (34.7)	20.43 (−0.34, 41.20)
Platelet count day 5, mean (SD), ×10^9^/L*	234.3 (31.9)	262.1 (31.1)	27.77 (10.38, 45.17)

* *P*-value < 0.05.

There were no differences in the WBC counts on days 3 and 5 and the platelet counts on day 3 between the two study groups. The mean platelet count on day 5 was greater in the bCPAP group compared to the MV-CPAP group (mean difference = 0.24; 95% CI: 10.38–45.17, *p* = 0.002) (shown in Table 2). There were no episodes of hyper- or hypothermia for any neonate in either group.

## 4 Discussion

This is the first study that sought to assess the potential risk of introducing nosocomial infections associated with use of cleaned and reprocessed bCPAP systems. Among the 75 randomized neonates no clinically significant differences were detected.

Two meta-analyses that assessed the risk of cross-contamination among reusable bronchoscopes found an 8.36% and 2.8% increase in risk of infection after reuse of bronchoscopes (20, 21). A study that evaluated the rates of microbial contamination in reusable humidifiers found that 60% of humidifiers in a NICU were contaminated with bacteria (22). In our study, no differences were detected in the odds of clinical deterioration or in the duration of CPAP treatment between the intervention and control groups.

The ideal indicators defining sepsis in neonates remain elusive (17, 23). A large multicenter study in the United States found that neonatal sepsis was associated with high (> 20.3 × 10^9^/L) or low (< 6.8 × 10^9^/L) WBC counts and thrombocytopenia (< 130 × 10^9^/L) (24). In our study, both groups had WBC counts and platelet counts within normal ranges (WBC counts: 5–20 × 10^9^/L, platelet counts: 150–450 × 10^9^/L) on days 1 through 5, and were not different from one another. The mean platelet count of the intervention group was greater than in the control group on day 5, however the difference was not likely clinically significant, especially since neonatal sepsis is typically associated with thrombocytopenia.

The safe use of cleaned and reprocessed medical devices requires training of healthcare workers on cleaning and disinfection procedures and strict adherence to established validated protocols that minimize infection risks (25–27). While strict compliance with safe reprocessing procedures is a challenge in all settings it may especially be so in low resource environments where reuse of medical devices is a matter of necessity, yet overcrowding, shortages of protective equipment, lack of clean water, and environmental pollution are common (28). Reuse of medical devices in resource constrained environments is critical to maximizing access to lifesaving interventions by addressing access barriers such as high cost and weak supply chains as well as minimizing medical waste. The findings from our study suggest that even with use of the ITRMC modified reprocessing protocol the bCPAP system appears safe for multiple patient uses after being cleaned and disinfected between each neonate.

There were a few limitations with our study. The first was the lack of consensus on the definition of neonatal sepsis (29). While there are some variations in the literature, we used the most commonly employed indicators. They included the clinical outcomes of death, need for escalation of treatment, clinical deterioration, and the laboratory markers of the WBC and platelet counts (30). A second limitation was that the study was a *post hoc* analysis of a previously performed randomized control trial. The criteria of the original study excluded the sickest of the neonates, therefore limiting the size of the two groups and the population vulnerability. It is possible that differences between the two groups would have been found with a larger sample size and with inclusion of the most critically ill neonates. Thirdly, the staff at ITRMC did not use the validated cleaning and disinfection protocol described in the Instruction for Use. However, the protocol they followed was a subset of the validated one, therefore would have potentially led to a higher risk of infection than the validated protocol. Lastly, the study included patients from only one facility which limits the generalizability of our findings. Since the quality of reprocessing depends heavily on protocol adherence, additional studies with a broader representation of facilities are needed.

This study showed that use of a reprocessed bCPAP system designed to increase global access to CPAP did not increase rates of neonatal sepsis. Further study in different settings will better define optimal safety in reuse of these bCPAP devices.

## Data Availability

The data analyzed in this study is subject to the following licenses/restrictions: all data is available upon request. Requests to access these datasets should be directed to sarahbadin480@gmail.com.
